# PARP1 and PARP2 are dispensable for DNA repair by microhomology-mediated end-joining at double-ended DSBs

**DOI:** 10.1093/nar/gkaf1437

**Published:** 2026-01-06

**Authors:** Raquel Ortega, Erin R Taylor, Sophie M Whitehead, Thomas Danhorn, Benjamin G Bitler, Nausica Arnoult

**Affiliations:** Department of Molecular, Cellular, and Developmental Biology, University of Colorado Boulder, Boulder, CO 80309, United States; Division of Reproductive Sciences, Department of Obstetrics and Gynecology, University of Colorado Denver, Anschutz Medical Campus, Aurora, CO 80045, United States; Department of Molecular, Cellular, and Developmental Biology, University of Colorado Boulder, Boulder, CO 80309, United States; Department of Molecular, Cellular, and Developmental Biology, University of Colorado Boulder, Boulder, CO 80309, United States; University of Colorado Cancer Center, University of Colorado Anschutz Medical Campus, Aurora, CO 80045, United States; Department of Biomedical Informatics, University of Colorado Anschutz Medical Campus, Aurora, CO 80045, United States; Division of Reproductive Sciences, Department of Obstetrics and Gynecology, University of Colorado Denver, Anschutz Medical Campus, Aurora, CO 80045, United States; Department of Molecular, Cellular, and Developmental Biology, University of Colorado Boulder, Boulder, CO 80309, United States

## Abstract

Poly ADP-ribose polymerase (PARP) inhibitors are standard of care treatment for cancers with homologous-recombination deficiencies. Yet, as tumours develop resistance, complementary strategies are emerging, including targeting microhomology-mediated end joining (MMEJ). Given that PARP1 is widely described as a key promoter of MMEJ, PARP1 inhibition should suppress MMEJ, making MMEJ inhibition redundant. MMEJ was first characterized as a backup repair pathway, with early studies linking PARP1 to MMEJ in nonhomologous end joining-deficient cells. However, we now recognize that MMEJ is active in cells with intact repair pathways, primarily operating during mitosis. The role of PARP1 in this context remains unclear. Here, we systematically examine how PARP1/2 and PARP inhibition affect MMEJ activity in cells with intact repair pathways. Surprisingly, PARP1/2 inhibition leads to elevated Polθ-dependent MMEJ levels at ISceI-mediated double-stranded DNA breaks (DSBs), an increase that is dependent on homologous recombination. We next show that MMEJ at double-ended DSBs mainly occurs during mitosis, with no detectable activity during G1. Importantly, we show that PARP1 and 2 are dispensable for MMEJ at double-ended DSBs (deDSBs) and is expendable for repair of DSBs during mitosis. Altogether, this data shifts the understanding of the role of PARP1 in MMEJ and DNA repair pathway choice and further strengthens a rationale for PARPi/MMEJi combinatorial drug treatment in HR-deficient cancers.

## Graphical Abstract

**Graphical Abstract:**
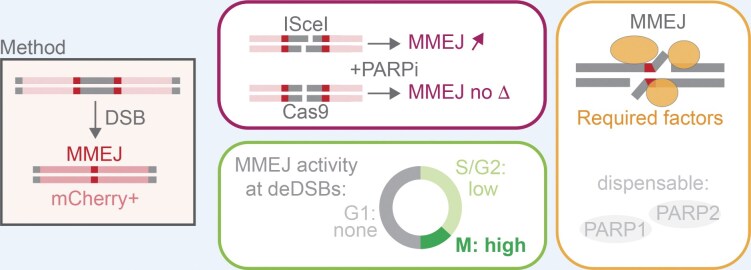

## Introduction

Double-stranded DNA breaks (DSBs) pose a major threat to genome integrity, as inaccurate DSB repair can result in significant chromosomal aberrations [1]. PARP1 (poly ADP-ribose polymerase 1) is a highly abundant nuclear protein and, among its roles in DNA damage repair [2–4], functions as an initial DSB sensor. DSB-binding activates PARP1, helping orchestrate the DNA damage response (DDR) and repair [3, 5, 6]. PARP1 uses nicotinamide adenine dinucleotide (NAD+) to polymerize poly (ADP)-ribose (PAR) onto many substrates, including itself, chromatin, and other DDR proteins [2, 4, 7]. PARP1, the most extensively studied member within the family of 17 related ADP-ribosyl transferases (ARTDs), accounts for up to 80%–90% of PARylation events [8]. However, PARP1, PARP2, and PARP3 have all been described as functioning in response to DNA damage [9].

PARP1-dependent vulnerability was first exploited clinically when it was found that homologous recombination-deficient (HRD) *BRCA1*- and *BRCA2*-null cells are uniquely sensitive to PARP inhibition [10, 11]. Since this seminal work, PARP inhibitors (PARPi) have become a first-line maintenance treatment for HRD cancers [12, 13]. PARPi (e.g. olaparib) functions by competing with NAD + for binding to both PARP1 and PARP2. PARPi are proposed to be lethal through multiple mechanisms, including PARP trapping, inhibiting PARP1/2 catalytic activity, increasing single-stranded DNA gaps, and destabilizing the replication fork machinery [10, 11, 14–21]. However, only 50% of HRD tumours respond to PARPi treatment due to intrinsic resistance, and, of those that respond, most patients eventually relapse due to acquired drug resistance [22–24]. PARPi resistance in HRD cancer cells can occur through a myriad of mechanisms, including restoration of homologous recombination (HR), DNA replication fork protection, diminished PARP trapping, PARPi efflux, chromatin remodelling, and, in the case of *BRCA1*-mutated cancers, *53BP1* mutation [16, 25–34]. Thus, overcoming PARPi resistance is a key priority and has ushered in the development of PARPi combination strategies to enhance treatment efficacy. Current efforts to combat resistance include PARPi combined with drugs that target cell cycle progression, immune checkpoint blockade, angiogenesis, and the DDR pathway [12]. Specifically, one DDR/PARPi combination approach targets the canonical polymerase of microhomology-mediated end joining (MMEJ), Polymerase Q (Polθ, encoded by *POLQ*), as Polθ is essential in HRD cells [35–38]. Notably, Polθ inhibition enhances the cytotoxicity of PARPi and, in some cases, even restores sensitivity to PARPi in PARPi-resistant cells [35, 38]. Currently, clinical trials are assessing the effectiveness of Polθi in combination with PARPi to treat HRD cancers [39]. Thus, understanding the interplay and mechanisms of action between MMEJ and PARP inhibitors is critical.

MMEJ is an intrinsically mutagenic DSB repair pathway. Mammalian MMEJ begins with initial resection at the break-site, involving Mre11/Rad50/Nbs1 (MRN) and CtIP [40, 41]. Resection leads to exposure and annealing of microhomologies flanking the break, allowing for synapsis of the ends through Polθ [39, 42, 43]. Microhomology annealing results in the formation of nonhomologous 3′ overhangs that are subsequently removed by nucleases Ape2 [44] or Xpf/Ercc1 [45]. Polθ’s polymerase domain can then use the microhomologies as primers to perform fill-in synthesis [36, 46, 47]. Polδ, Polλ, and Polβ have also been shown to perform this function in varying contexts [43, 48–50]. Finally, the DNA ends are ligated by Lig3-Xrcc1 or Lig1 [51–54]. The essentiality of PARP1 in regulating MMEJ, however, is controversial.

PARP1 was first identified to promote mammalian MMEJ through *in vitro* DNA pulldown assays demonstrating that PARP1 can mediate DNA synapsis at overhangs in HeLa cells, independently of the canonical nonhomologous end joining (NHEJ) proteins, DNA-PKcs and Ku70/80 heterodimer [55]. Later, the Ku70/80 complex was shown to compete with PARP1 for DNA binding [56]. PARP1 promotion of MMEJ repair was then further demonstrated using other cell-free methods [57, 58]. In NHEJ-deficient cells, PARP1 promotes MMEJ during class switch recombination [59] and chromosomal translocations [60]. Moreover, at deprotected *XRCC5*^−/−^ (Ku80) mouse telomeres, we and others have observed that MMEJ-dependent telomere fusions are abrogated when treated with PARPi [44, 61]. In mammalian cells, PARPi and PARP1 knockdown lead to reduced Polθ recruitment to irradiation sites [36, 62]. Altogether, these data suggest that the role of PARP1 in MMEJ may be to promote DNA synapsis and to recruit Polθ to DSBs. However, it is important to note that much of the data regarding PARP1 role in MMEJ was collected in interphase and/or in the absence of canonical NHEJ factors that physiologically compete with MMEJ.

MMEJ strongly competes with NHEJ in G1 and with NHEJ and HR in S/G2 phases [26, 37, 40, 63–66]. Meanwhile, MMEJ is a highly active DSB repair pathway in mitosis, where NHEJ and HR are actively inhibited [67–73]. For example, it has been described that in the absence of *BRCA2*, replication-induced DSBs are repaired by MMEJ in mitosis [74]. Moreover, two recent publications showed that Polθ activity and recruitment to DSBs are highly dependent on mitotic-specific factors [72, 73]. These data suggest that mitosis is possibly the cell cycle phase when MMEJ is most active and physiologically relevant. Considering mitotic-specific recruitment of Polθ to DSBs, it is unclear what role PARP1 plays in MMEJ in cycling cells when all DSB repair pathways are active and competing.

Utilizing a DNA repair reporter, we assessed MMEJ levels following treatment with various PARP inhibitors, including those with minimal PARP trapping activity. Remarkably, PARP inhibition systematically led to elevated *POLQ*-dependent MMEJ levels in cells with intact repair pathways. We further found that MMEJ upregulation is mediated by inhibition of PARP1, not PARP2, and is dependent on HR. We show that MMEJ at double-ended DSBs (deDSBs) primarily occurs during mitosis, with low levels of activity in S/G2, but none in G1. Finally, PARP1 and 2 are dispensable for MMEJ at double-ended DSBs and for repair of DSBs occurring in mitosis. Overall, our findings challenge the current paradigm by demonstrating that PARP1 does not promote MMEJ. These results have important implications for the development of combination therapies using MMEJ and PARP inhibitors to treat HRD cancers.

## Materials and methods

### Cell lines, cell culture, and chemical compounds

Fibrosarcoma HT1080 cells were purchased from ATCC (CCL-121). MEFs TRF2^F/−^ Rosa26-CreERT2 were established in the de Lange lab and obtained from ATCC (CRL-3317). Lenti-X 293T cells were derived from a transformed human embryonic kidney cell line and purchased from Takara (#632180). Cell lines and their derivatives were grown in Dulbecco’s modified Eagle’s medium (DMEM) medium (Corning #10-013CV) with 10% Cosmic Calf Serum (Cytiva HyClone SH30087.04), 100 mg/ml penicillin/streptomycin L-Glutamine (Corning #30-009-CI), and 1× MEM Nonessential Amino Acids (Corning #25-025CI). PEO1 olaparib-resistant (PEO1-OR) cells were created by dose-escalating olaparib in PEO1 (*TP53/BRCA2*-mutated) cells [30] which were obtained from the Gynecologic Tissue and Fluid Bank (GTFB) at the University of Colorado and authenticated at the University of Arizona Genomics Core using short tandem repeat DNA profiling [30]. PEO1-OR cells were maintained in RPMI 1640 supplemented with 10% Cosmic Calf Serum (Cytiva HyClone SH30087.04) and 100 mg/ml penicillin/streptomycin L-Glutamine (Corning #30-009-CI). All cell lines were maintained at 3% O_2_ and 7.5% CO_2_.

*Chemical compounds used include:* dimethyl sulfoxide (DMSO) (1%, Thermo Fisher, #J66650.AP), olaparib (500 nM–20 µM, Selleckchem, AZD2281 #S1060), DNA-PKcsi (1 µM in HT0180 cells and 2.5 µM in PEO1-OR cells, NU7441, R&D Systems #3712/10), and Polθi (10 µM, ART558, MedChemExpress Cat. No.: HY-141520), nocodazole 100 ng/ml (MedChemExpress, #HY-13530), RO3306 9 µM (MedChemExpress, #HY-12529), pablociclib 250 nM (Med Chem Express, HY-50767C).

### Plasmids

The MMEJ reporter (pLenti-Puro-MMEJ_rep) used in experiments was described previously [44]. The ISceI-BFP plasmid to cut the MMEJ reporter was derived from the pCBASceI plasmid, obtained from Maria Jasin [75] (Addgene #26477) in which nucleotides 1730–2587 have been replaced by codon optimized ISceI-T2A-BFP. The Cas9-GFP plasmid to cut the MMEJ reporter was derived from pSpCas9(BB)-2A-GFP plasmid (obtained Feng Zhang [76], Addgene #48138), and golden gate was used to insert either the nontargeting guide (CTCGACAGTTCGTCCCGAGC) or a guide against the MMEJ reporter (CTGCAAGATTAGGGATAACA). The GFP-PARP1 plasmid used to complement *PARP1*-KO clones was a generous gift from the Karolin Luger lab and was derived by cloning the human *PARP1* coding region into the plasmid pEGFP-C3 (Clontech #6082-1). The *GFP-PARP1* sequence was then inserted into the lentiviral backbone of pLenti-Puro-MMEJ_rep where the MMEJ reporter was removed from bp 4121 to 5641 and replaced with *GFP-PARP1*, and then Puromycin selection was replaced with Hygromycin B selection from bp 6226 to 6775. Site-directed mutagenesis was then used to mutate the PAM sequence of the plasmid corresponding to the gRNA used to knock out *PARP1*. The *PARP2* plasmid used to complement *PARP2*-KO clones was a generous gift from the Karolin Luger lab and was derived by cloning the human *PARP2* coding region into plasmid mCherry C1 (obtained from Dyche Mullins [77], Addgene #58476). The *PARP2* sequence was then inserted into the lentiviral backbone of pLenti-Puro-MMEJ_rep where the MMEJ reporter was removed from bp 4121 to 5641 and replaced with *PARP2*, and then puromycin selection was replaced with Hygromycin B selection from bp 6226 to 6775. Site-directed mutagenesis was then used to mutate the PAM sequence on the plasmid corresponding to the gRNA used to knock out *PARP2*. The Cas9 plasmid used to knockout genes of interest was derived from TLCV299 (a gift from Adam Karpf [78], Addgene #87360). This plasmid was modified by removing eGFP and replacing puromycin selection with blasticidin selection. sgRNAs were cloned using golden gate. The Cas9-CtIP construct was obtained from Jean-Paul Condorcet (Addgene #109403) [79] and cloned in a plasmid expressing a guide RNA targeting the MMEJ reporter. All plasmids were verified by full plasmid nanopore sequencing.

### Lentiviral transductions

Lenti-X 293T cells (550000) were seeded into one well of a six-well plate. Twenty-four hours later, 2 ml complete DMEM media with 10 mM HEPES was replenished on cells. Cells were then immediately transfected with packaging plasmids (0.5 μg pCMV-VSV-G, 1.6 μg pD8.9), 1.7 μg transfer plasmid, and 11.1 μg of PEI MAX (Polysciences Inc., #24765-1) in 500 μl Opti-MEM (Gibco, #31985070). Media was changed after 24 h with 3 ml of complete DMEM. Virus-containing supernatant was collected 48 and 72 h post-transfection, filter sterilized on a 0.45 mm PES Filter membrane (Whatman Uniflo, #9915-2504) and directly used to infect cells in the presence of 5 mg/ml polybrene (EMD Millipore, #TR-1003-G). Forty-eight hours after infection, cells were washed and selected with 1 mg/ml puromycin (Alfa Aesar, #J61278-MC, 200 mg/ml Hygromycin B (Biosciences, #31282-04-9), or 10 mg/ml blasticidin (RPI, #B12200-0.05). Selection was performed until complete death of uninfected control cells.

### Establishment of knock-out cell lines

For knockout of human *PARP1, PARP2, EXO1, WRN*, and *53BP1*, HT1080 cells were transduced with pLentiGuide-Cas9-Blast and seeded for clones. For *BRCA2* and *RHNO1*, 1 million cells were mixed with 4 µM of guide RNAs (synthesized from Synthego) and 2µM of Cas9 (IDT DNA, #326154273), electroporated with a MaxCYTE electroporator (E-TAP-Atx, MaxCyte) according to the manufacturer’s protocol, and seeded for clones. The single guide RNA sequences for each gene knockout are as follows: PARP1 (CGATGCCTATTACTGCACTG), PARP2 (TTGTTCAGGCAATCTCAACA), *BRCA2* (AGCACAGUAGAACUAAGGGU), *EXO1* (TCAGGGGGTAGATTGCCTCG), WRN (ATCCTGTGGAACATACCATG), and *53BP1* (TCATGTGACGATGTAAGACA), *RHNO1* (UUCGACUUGAGUGUCUCGCC). The nontargeting guide (CTCGACAGTTCGTCCCGAGC) was included as a Cas9 control. Guide cloning was done following the golden gate cloning protocol and verified by Sanger sequencing. Clonal lines were isolated and clonal knockout verified by immunoblot.

### MMEJ and DR-GFP ISceI/Cas9 fluorescent reporter assay

MMEJ reporter assay: HT1080 cells with or without *PARP1*^−/−^, *PARP2*^−/−^, *EXO1*^−/−^, or *WRN*^−/−^ were transduced with pLenti-Puro_MMEJ_rep and selected with puromycin. Cells were seeded onto 12-well plates 24 h before adding either ISceI or Cas9. Cells were seeded such that they would be 80%–90% confluent 24 h later. Then, cells were transfected with either ISceI-T2A-BFP or Cas9-T2A-GFP-guide1. Transfections were done with lipofectamine 3000 (Invitrogen, #L3000015) using 1 µg DNA, 2 µl p3000, and 2 µl lipofectamine 3000 per reaction (following manufacturer’s instructions). 5 h later, cells were split 1/3 into six-well plates and drugs were added. At day 3, cells were analysed by flow cytometry (MACS Quant VYB, Miltenyi Biotec) for BFP and mCherry expression. BFP+ (ISceI+) or GFP+ (Cas9+) cell populations were gated and mCherry + cells were quantified.

MMEJ assay in G1: Cells were plated at 60% confluence into six-well plates. Eight hours later, Palbociclib was added at a final concentration of 250 nM. Cells were transfected 16 h later with 2.25 µg of DNA, 4.5 µl of p3000, and 4.5 µl of lipofectamine 3000. Drugs were added 5 h after transfection, and flow cytometry was performed 48–56 h post-transfection.

DR-GFP assay: HT1080 cells were transfected with the DR-GFP plasmid (obtained from Maria Jasin [75], Addgene #26477) and cells with stable integration were selected with puromycin. Transfection of ISceI or Cas9 (with a guide RNA targeting the DR-GFP sequence—GTGTCCGGCTAGGGATAACA) and flow cytometry analysis were carried out as described for the MMEJ reporter.

### Amplicon sequencing of MMEJ reporter

HT1080 cells were transfected with ISceI-T2A-BFP. Five hours later, cells were treated with DMSO (0.1%), Polθi (ART558, 10 µM), or olaparib (5 µM). At day 3, cells were harvested. Genomic DNA was extracted using QuickExtract (Biosearch Technologies, #QE09050). One PCR reaction was done on each condition using Illumina MiSeq adapter sequences linked to primers that amplify a 78 bp sequence around the ISceI cut region of the reporter. Amplification was done in 50 µl reactions with Q5 High-Fidelity 2X Master Mix (New England Biolabs, #M0492S), 1 µg template, and 0.25 µM of primers for 30 cycles. Products were purified using gel extraction of the bands in 0.6% agarose gel. Using manufacturer instructions, concentration was calculated using TapeStation 4150 (Agilent, #G2992AA). Libraries were pooled at 2.5 nM and sequenced at the University of Colorado Genomics Shared Resource (RRID:SCR_021984). NovaSeq 6000 sequencing system was used, with paired end reads of 2 × 150 bp for 50 million reads. Raw sequences were analysed for DNA repair products at the University of Colorado Anschutz Medical Campus Cancer Center Bioinformatics Core Facility (RRID:SCR_021983). The overlapping forward and reverse raw sequence reads were combined into end-to-end sequences of fragments with NGmerge version 0.3 [80], allowing a mismatch fraction of 0.1. The adapter trimming software skewer version v0.2.2 [81] was used to remove the sequence TTTCCGGAAGGGTTCAAGTGGGAGAGGGTAATGAATTTTGAGGATGGCGGGGTTGTCACCGTTA from the 5′-end with parameters “-m head -Q 20 -r 0.1 -d 0.05 -n -l 20″ and the sequence ACAAAGTGAAGTTGCGAGGAACAAATTTCCCAAGCGA from the 3′-end with parameters “-m tail -Q 20 -r 0.1 -d 0.05 -n -l 10 -L 70″ in order to focus the analysis on the relevant part of the fragment sequences. Low-quality sequences were removed using the fastq_quality_filter tool from the FastX toolkit version 0.0.14 [https://github.com/agordon/fastx_toolkit] with parameters “-q 20 -p 100″. Sequences that did not possess the expected 5′- and 3′-ends were filtered out with seqkit grep version 2.6.1 with parameters “-s -r -P -p ‘^GCTT|AGC(?:GA?)?$’”. Identical sequences were enumerated using the fastx_collapser tool from the FastX toolkit.

### Immunoblotting

Cells were collected and washed once using cold phosphate buffered saline (PBS). Protein was extracted using Radioimmunoprecipitation Assay buffer (RIPA) buffer (50 mM Tris–HCl, pH 8, 1% NP-40, 0.5% sodium deoxycholate, 0.1% sodium dodecyl sulphate, 150 mM NaCl) combined with protease and phosphatase inhibitor cocktail (Roche, #05892791001) and 1:100 benzonase (Millipore, #70746). Protein concentration was measured using the bicinchoninic acid (BCA) protein assay (Thermo Fisher Scientific, #23227). A total of 25 µg of protein extract was separated on NuPAGE Bis–Tris 4%–12% gels (Invitrogen, #WG1402BOX) and transferred onto a Polyvinylidene Fluoride (PVDF) membrane (Cytiva, #GE10600058). Membrane was blocked by shaking 1 h at room temperature in 5% bovine serum albumin (BSA) in PBS-Tween (PBST). The membrane was incubated overnight, rocking at 4°C with primary antibodies diluted in 5% BSA in PBST. The next day, the membrane was washed three times with PBST, incubated for 1 h at room temperature in secondary antibody diluted in 5% BSA in PBST, and washed three times with PBST. Proteins were detected using ProSignal Fempto (Prometheus, #20-302). Blots were imaged using G:Box chemi XX6 (Syngene).

*Antibodies used for immunoblotting:* PARP1 (diluted 1:2000, Cell Signaling Technology #9542), PARP2 (diluted 1:2000, Active Motif #39744, Antibody has been discontinued), β-Actin (diluted 1:5000, Cell Signaling Technology #8457), WRN (diluted 1:2000, Cell Signaling Technology #4666, RRID:AB_10692114), EXO1 (diluted 1:1000, Cell Signaling Technology #63862), PAR (diluted 1:2000, Cell Signaling Technology #87733), 53BP1 (diluted 1:2000, Novus Biologicals #NB100-304), H3 (diluted 1:5000, Cell Signaling Technology #9715), anti-mouse IgG, HRP-linked (diluted 1:5000, Cell Signaling #7076), anti-rabbit IgG, HRP-linked (diluted 1:5000, Cell Signaling #7074), BRCA2 (diluted 1:1000, Bethyl #A303-434A, RRID:AB_10952240).

### Propidium iodide and pH3S10 staining

Protocol from [82] was used. Briefly, cells were trypsinized, spun down, and washed in PBS. Cells were fixed with 70% ethanol and kept at −20°C until use. Cells were spun down, washed in PBS, and resuspended in 1.4 ml PBS with 0.25% Triton X-100 on ice for 15 min. Cells were washed and resuspended in 100 μl of PBSBA [PBS containing 1% BSA (w/v)] with 0.75 μg anti-phosphorylated histone H3 serine 10 (pH3S10), Alexa Fluor 488 Conjugate (Millipore Sigma, #06-570-AF488) on a rocker, protected from light for 3 h. Cells were then spun down, washed in PBSBA, spun down again, and resuspended in 220 μl propidium iodide (PI) solution (500 μg/ml RNAse (Thermo Fisher Scientific, #EN0531), PI 100 μg/ml in PBS) for 30 min at 37°C. Cells were then analysed by flow cytometry (MACS Quant VYB, Miltenyi Biotec) to identify FITC + cells and PI + cells.

### Immunofluorescence of DSBs during mitosis

Cells were seeded on glass coverslips and, once attached, treated with 9 µM RO3306 for 16 h. After 15-h treatment, DMSO (0.1%), olaparib (5 µM), or Polθi (ART558, 10 µM) were added. Cells were then washed with PBS and released into fresh media containing 10 ng/ml nocodazole as well as DMSO (0.1%), olaparib, or Polθi. Forty minutes after release, cells were irradiated at 2 Gy using a Faxitron Cabinet X-ray System Model RX-650 and placed back into the incubator for 1 or 5 h. Cells were then fixed with 3.7% formaldehyde in 1× PBS, washed, and permeabilized 10 min with KCM buffer (120 mM KCl, 20 mM NaCl, 10 mM Tris, pH 7.5, 0.1% Triton), and stored in PBS at 4°C until immunostaining. Cells were blocked 30 min at 37°C with ABDIL (20 mM Tris, pH 7.5, 2% BSA, 0.2% Fish Gelatin, 150 mM NaCl, 0.1% Triton, 0.1% sodium azide), incubated 1.5 h at 37°C with primary antibodies diluted in ABDIL. Cells were then washed three times with PBST and incubated 1 h at room temperature with secondary antibodies, washed again three times in PBST and fixed with Fluoroshield containing DAPI (Sigma–Aldrich, #F6057). Imaging was acquired using a NikonTie SDC at 20× magnification through the University of Colorado Light Microscopy Core Facility (RRID:SCR_018993). Image processing and analysis were performed using FIJI (ImageJ) with a custom macro. For each field of view, Z-stacks were acquired, and a sum projection was applied to maximize signal detection. Mitotic cells were identified based on pH3S10 positivity, and regions of interest (ROIs) were manually outlined around mitotic nuclei. Integrated density values for γH2A.X foci were measured within the defined mitotic ROIs.

*Antibodies used for immunofluorescence (IF)*: Phosphorylated γH2A.X (Ser139) (diluted 1:5000, EMD Milipore Cat#05-636), phosphorylated H3S10 (diluted 1:1000, Cell Signaling Technology #9701), goat anti-mouse IgG (H + L) cross-absorbed secondary antibody, Alexa Fluor 568 (diluted 1:400, Thermo Fisher Scientific #A11004), goat anti-rabbit IgG (H + L) cross-adsorbed secondary antibody, Alexa Fluor 488 (diluted 1:400, Thermo Fisher Scientific #A11008).

### Analysis of micronuclei

Cells were seeded on glass coverslips and treated with 9 µM RO3306 for 16 h. After 15 h of treatment, DMSO (0.1%), olaparib (5 µM), or Polθi (ART558, 10 µM) were added. Cells were then washed with PBS and released into fresh media containing again DMSO, olaparib, or Polθi. Forty minutes after release, cells were irradiated at 2 Gy using a Faxitron Cabinet X-ray System Model RX-650 and allowed to recover for 5 h. Cells were then fixed with 3.7% formaldehyde in 1× PBS, washed, and coverslips were mounted with fixed with Fluoroshield containing DAPI (Sigma–Aldrich, Cat #F6057). Cells with or without micronuclei were counted using an Echo Revolve Light Microscope, using a 40× objective. Representative images were taken using the same conditions.

### Statistics

Graphs and statistical analysis were completed in Prism GraphPad (v9). Data are presented as mean ± standard error of the mean (SEM). An unpaired Student’s *t* test was used for statistical comparison between control and treatment group. A one-way Analysis of variance (ANOVA) was used to determine variance among multiple gestational groups, with a Benjamini–Hochberg multiple comparison *post-hoc* test to determine significance between individual groups. A *P*-value of <.05 was considered significant.

## Results

### PARP1/2 inhibition increases MMEJ at ISceI-mediated breaks

**Figure 1.**
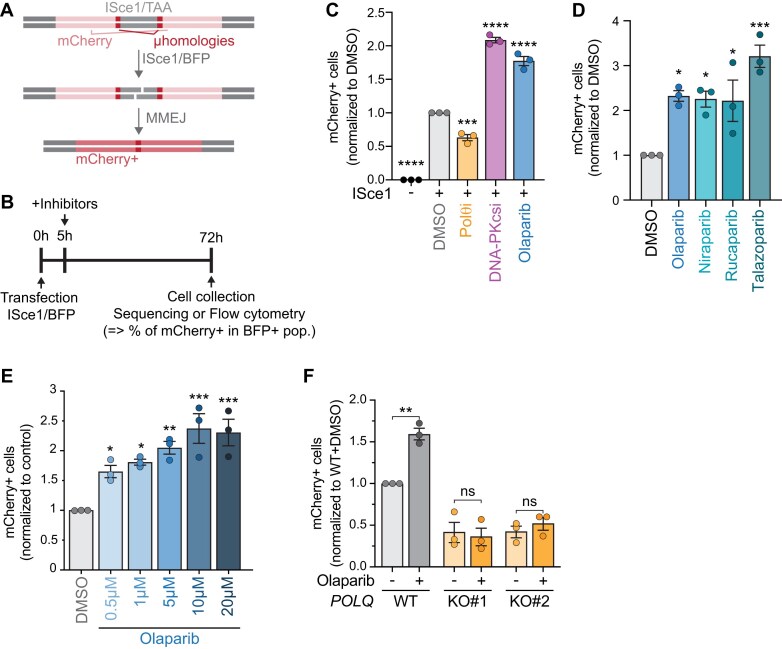
PARP inhibitors increase MMEJ at ISceI-mediated deDSBs. (**A, B**) Schematic (**A**) and experimental timeline (**B**) of MMEJ reporter used in panels (C)–(F), Figs 2C and D, 3A, C, and E, 5C and D, and 6B and C. Cutting the reporter with ISceI and repair by microhomology annealing to an in-frame, functional mCherry gene. Flow cytometry was used to identify the mCherry + cells (MMEJ+) within the BFP+ (ISceI+) population. (**C–E**) MMEJ quantification using reporter and timeline from panel (A) and (B) in HT1080 cells. Values are normalized to DMSO. Drugs used were olaparib (5 µM or as indicated), DNA-PKcsi (NU7441, 1 µM), Polθi (ART558 10 µM), niraparib (2.5 µM), rucaparib (2.5 µM), and talazoparib (2.5 µM). (**F**) MMEJ quantification in HT1080 parental cells compared to isogenic *POLQ*-KO cells following olaparib treatment. Values are normalized to wild-type DMSO. Statistical analyses for panels (C)–(F): Data represent three independent experiments, each the average of three technical replicates. Data are mean ± SEM. Statistical test, one way ANOVA with multiple comparison correction. ns: nonsignificant, *P <.05, **P <.01, ***P <.001, ****P <.0001.

PARP1 catalytic activity is described as essential for MMEJ activity, and PARP1 can be effectively inhibited pharmacologically using PARP1/2 inhibitors (PARPi), with olaparib being the most widely studied [83]. Thus, we wanted to examine the MMEJ repair response in the presence of olaparib. To measure MMEJ activity, we used a fluorescent genetic reporter that we previously described [44], consisting of an integrated mCherry cassette with an internal ISceI restriction site flanked by 10 bp microhomologies. The reporter is designed to enable mCherry fluorescence only when cut and repaired by annealing of the 10 bp microhomologies (Fig. 1A). We transduced the reporter into HT1080 fibrosarcoma cell lines at an MOI (multiplicity of infection) of 0.3 to integrate one copy per antibiotic-selected cell. The integrated reporter can then be cut by transiently transfecting a plasmid that expresses ISceI as well as BFP, allowing the identification of the transfected population.

Five hours after ISceI transfection, we treated cells with PARP, Polθ, or DNA-PKcs inhibitors, and analysed them by flow cytometry three days later (experimental timeline depicted in Fig. 1B). We chose this time point for drug addition to avoid the potential impact of the drugs on transfection efficiency, while reasoning that DSBs would not yet have occurred, as ISceI expression is still minimal at 5 h (Supplementary Fig. S1A). The frequency of mCherry + cells within the BFP + population was used to measure MMEJ activity. Importantly, all conditions had similar levels of BFP + cells, indicating that transfection levels were similar in all conditions (Supplementary Fig. S1B). As expected, we found no mCherry-positive cells in the control population not transfected with ISceI (Fig. 1C). Likewise, inhibiting the primary MMEJ polymerase, Polθ (Polθ inhibitor ART558) [35] led to decreased MMEJ activity, while inhibition of the competing pathway, NHEJ (DNA-PKcs inhibitor NU7441), increased MMEJ activity (Fig. 1C). Finally, we treated cells with 5 μM olaparib, the lowest dose at which PARylation was fully ablated (Supplementary Fig. S1C) and which is a physiological dose [84]. Strikingly, we found a significant increase in MMEJ (Fig. 1C), a surprising result considering that PARP1 is described to promote MMEJ. To confirm this result, we complemented the flow-based assays with amplicon sequencing and found higher percentages of MMEJ repair products in olaparib-treated cells compared to untreated cells (Supplementary Fig. S1D and E). We also verified that the increase in MMEJ was not specific to olaparib and could be observed with other PARP inhibitors. Indeed, the results showed elevated MMEJ upon treatment with all the PARP inhibitors we tested (Fig. 1D and Supplementary Fig. S1F). Moreover, we observed a similar increase in MMEJ when we pre-treated cells with olaparib 24 h prior to ISceI transfection, rather than 5 h after (Supplementary Fig. S1G).

Since MMEJ activity varies over the cell cycle [40, 85], we tested whether PARPi led to cell cycle perturbation. As previously reported [86], olaparib treatment induced a dose-dependent increase in S and G2 phases and a decrease in mitotic index (Supplementary Fig. S1H and I). We, therefore, tested escalating doses of olaparib in our MMEJ reporter and found that all tested doses increased MMEJ levels, independently of their effect on the cell cycle (Fig. 1E). These data suggest that the observed increase in MMEJ is not a consequence of cell cycle perturbation. Of note, it is possible that the effect of higher doses of olaparib on MMEJ would be more prominent if the experimental timeline accounted for population doubling differences.

### PARPi-mediated MMEJ increase is dependent on Polθ

While Polθ is the primary polymerase responsible for MMEJ repair [36, 37, 87, 88], data show that other polymerases, such as Polλ [49], can also perform MMEJ repair. Thus, we wanted to determine whether the observed increase in MMEJ upon PARPi treatment depends on the canonical MMEJ polymerase. We measured MMEJ repair in olaparib-treated wild-type and isogenic *POLQ*-KO HT1080 cells [44]. As expected, *POLQ*-KO cells had significantly lower MMEJ levels than wild-type cells (Fig. 1F). Furthermore, we found that olaparib-mediated effects on MMEJ were abrogated upon *POLQ*-KO, demonstrating that the PARPi-dependent increase in MMEJ is *POLQ*-dependent. Similarly, a pharmacologic approach to inhibit Polθ activity attenuated the PARPi-dependent increase of MMEJ levels (Supplementary Fig. S2A), indicating that MMEJ increases seen upon olaparib treatment depend on the catalytic activity of Polθ.

Polθ activity during MMEJ repair has been reported to depend on both PARP1 and PARG [89]. To test whether PARG inhibition influences MMEJ at our reporter, we treated cells with escalating doses of the PARG inhibitor PDD. However, MMEJ activity remained unchanged across all drug concentrations (Supplementary Fig. S2B and C).

### PARPi-mediated increases in MMEJ are dependent on PARP1, not PARP2

**Figure 2.**
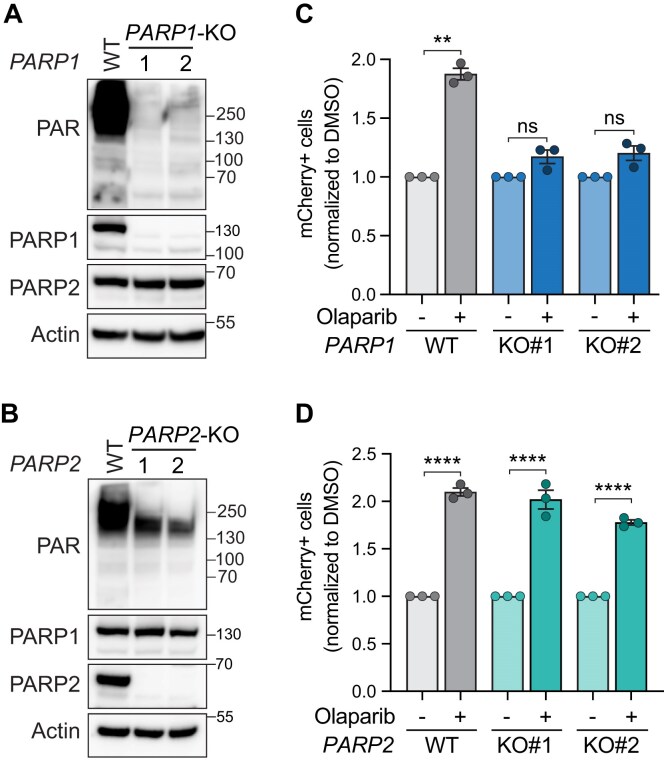
PARPi-mediated MMEJ increase is dependent on PARP1, not PARP2. (**A, B**) Immunoblot of Actin, PARP1, PARP2, and PAR in parental HT1080 cells and isogenic *PARP1*-KO (**A**) and *PARP2*-KO (**B**) clones. (**C, D**) MMEJ quantification using reporter and experimental timeline from Fig. 1A and B in HT1080 parental cells and indicated isogenic *PARP1*-KO (**C**) or *PARP2*-KO clones (**D**). Values are normalized to each cell line DMSO. Drug used was olaparib (5 µM). Statistical analyses for panels (C) and (D): Data represent three independent experiments, each the average of three technical replicates. Data are mean ± SEM. Statistical test, one-way ANOVA with multiple comparison correction. ns: nonsignificant, **P <.01, ****P <.0001.

Olaparib targets both PARP1 and PARP2 [90, 91]. To gain more mechanistic insight into what causes the MMEJ increase upon olaparib treatment, we wanted to determine whether it was due to inhibition of PARP1, PARP2, or both. We established isogenic *PARP1*-KO and *PARP2*-KO HT1080 clonal cell lines and measured the effect of olaparib on MMEJ. *PARP1* or *PARP2* knockouts were validated via immunoblot (Fig. 2A and B). As expected, *PARP1*-KO cells, similarly to olaparib-treated cells, showed almost complete ablation of PARylation (Fig. 2A). Next, we quantified MMEJ levels of *PARP1*-KO and *PARP2*-KO cells upon olaparib treatment. Compared to wild-type cells, olaparib treatment failed to increase MMEJ levels in *PARP1*-KO clones, demonstrating that PARP1 is required to mediate the olaparib-dependent increases in MMEJ levels (Fig. 2C). Conversely, *PARP2*-KO cells showed increased MMEJ levels with olaparib treatment, similarly to wild-type cells (Fig. 2D). Altogether, this data implicates catalytic inhibition of PARP1, not PARP2, in specifically modulating DNA repair towards MMEJ.

### PARPi-mediated MMEJ increase depends on HR

Next, we sought to determine the mechanism by which PARPi can lead to increased MMEJ following ISceI transfection. Since PARP1 was shown to promote Polθ recruitment and MMEJ repair in certain context [36, 44, 56, 92, 93], it is unlikely to directly inhibit MMEJ activity. Instead, we hypothesized that the observed increase in MMEJ is due to the suppression of a competing repair mechanism upon PARP1 inhibition.

**Figure 3.**
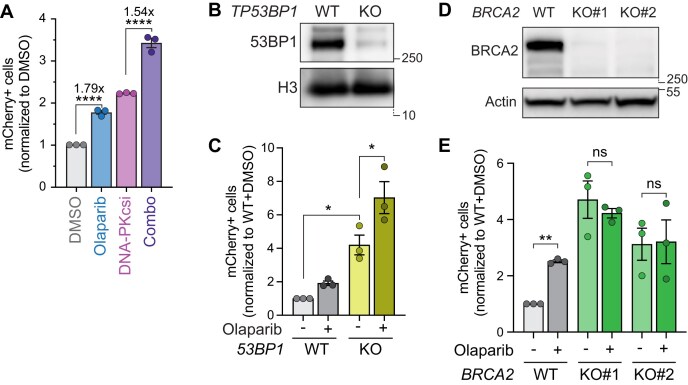
PARPi-dependent MMEJ increase depends on HR. (**A**) MMEJ quantification using the reporter and experimental timeline from Fig. 1A and B in HT1080 cells following treatment with olaparib (5 µM), DNA-PKcsi (NU7441, 1 µM), or both (combo). Values are normalized to DMSO. (**B**) Immunoblot of 53BP1 and histone H3 in parental HT1080 cells and isogenic *TP53BP1*-KO cells. (**C**) MMEJ quantification in HT1080 parental cells and indicated isogenic *53BP1*-KO cells. Values are normalized to wild-type DMSO. Drug used: olaparib (5 µM). (**D**) Immunoblot of BRCA2 and actin in parental HT1080 cells and two isogenic *BRCA2*-KO clonal lines. (**E**) MMEJ quantification in HT1080 parental cells and *BRCA2*-KO clones. Values are normalized to wild-type DMSO. Drug used: olaparib (5 µM). Statistical analyses for panels (A), (C), and (E). Data represent three independent experiments, each the average of three technical replicates. Data are mean ± SEM. Statistical test, one way ANOVA with multiple comparison correction. ns: nonsignificant, *P <.05, **P <.01, ****P <.0001.

PARPi and DNA-PKcsi have a similar effect on MMEJ (Fig. 1C), suggesting that PARP1 could be promoting NHEJ. We, therefore, tested whether DNA-PKcs inhibition and olaparib were epistatic; however, we found that the drug combination has an additive effect on MMEJ (Fig. 3A). Similarly, knockout of *53BP1* led to an increase in MMEJ activity, but treatment with olaparib was additive (Fig. 3B and C).

To determine whether, on the other hand, PARPi increases MMEJ by suppressing HR, we generated *BRCA2-*knockout cells (Fig. 3D) and measured MMEJ activity in this context. As expected, we found that MMEJ levels were higher when the competing pathway is suppressed. However, treatment with olaparib failed to further increase MMEJ in *BRCA2-*KO cells (Fig. 3E). These results indicate that PARPi likely prevents a subset of breaks from being repaired by HR, thereby redirecting them toward MMEJ repair.

PARP1 is proposed to influence long-range resection, although its precise role remains debated [94]. To test whether specific resection factors mediate the regulation of the HR/MMEJ balance by PARP1, we suppressed the long-range nuclease EXO1 and the DNA2 partner WRN, as DNA2 is essential and cannot be knocked out [95]. While loss of EXO1 or WRN each led to increased MMEJ, both knockouts still showed a further rise upon olaparib treatment (Supplementary Fig. S3A–D), indicating that neither EXO1 nor WRN alone account for the effect of PARP1 on the HR/MMEJ balance. However, we cannot exclude the possibility that PARP1 influences other long-range resection factors, such as DNA2 or BLM, or exerts redundant effects across multiple factors.

Alternatively, it is possible that PARPi does not directly inhibit HR, but rather generates a large number of single-ended DSBs that overwhelm HR capacity and divert repair factors away from the ISceI-induced breaks, which then rely on MMEJ.

Because the increase in MMEJ depends on HR, we did not expect PARP1/2 inhibition to modulate MMEJ in HR-deficient cancer cells. However, given the therapeutic implications, we tested this in BRCA2-mutated PEO1 cells that had acquired resistance to olaparib through prolonged drug escalation (PEO1-OR) [30]. Strikingly, MMEJ levels increased despite the absence of BRCA2 (Supplementaary Figure S3 E), indicating that PARP1/2 inhibition can still drive an MMEJ increase in HR-deficient cells. In this context, combined treatment with olaparib and DNA-PKcsi produced no additive effect (Supplementaary Figure S3 F), suggesting that the increase in MMEJ here stems from NHEJ inhibition. This data suggests that adaptation to chronic olaparib exposure may in some contexts rewire the regulation of repair pathway activity and modulation by PARP1.

### PARPi-dependent MMEJ increase does not occur at Cas9-induced breaks

**Figure 4.**
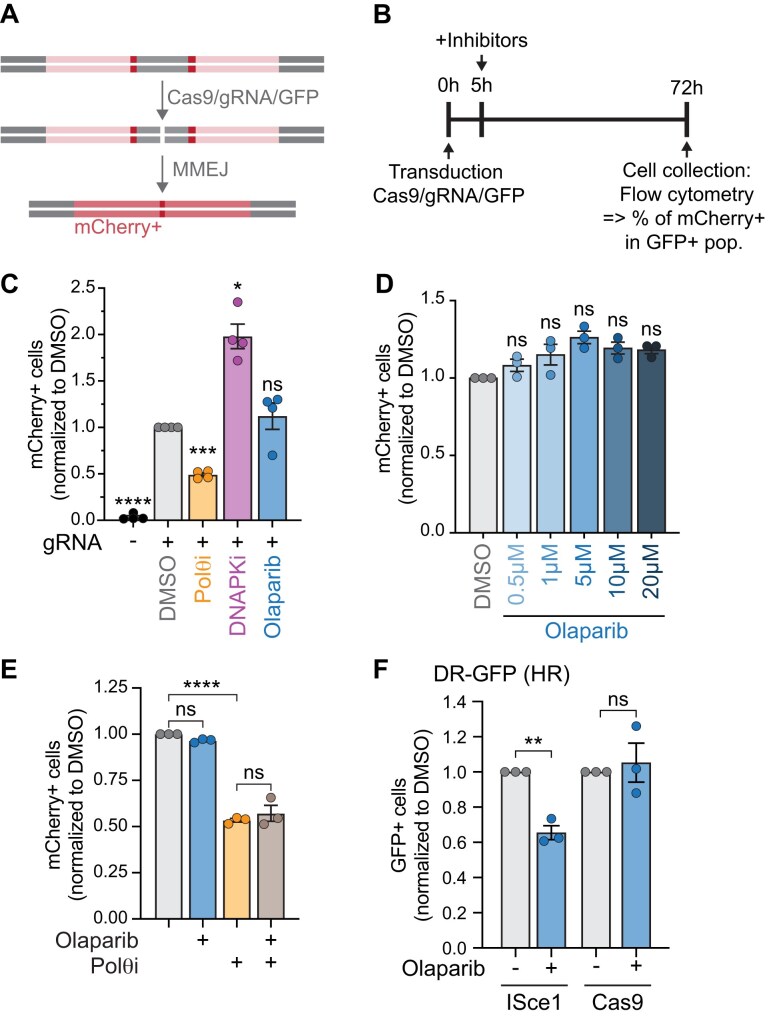
PARPi-dependent MMEJ increase does not occur at Cas9-induced DSBs. (**A, B**) Schematic (**A**) and experimental timeline (**B**) of MMEJ reporter used in panels (C)–(E), Figs 5E and 6B. Repair of the Cas9-induced cut with microhomology annealing leads to an in-frame, functional mCherry gene. Flow cytometry was used to identify the mCherry + cells (MMEJ+) within the GFP+ (Cas9+) population. (**C–E**) MMEJ quantification using the reporter and timeline from panels (A) and (B) in HT1080 cells. Values are normalized to DMSO. Drugs used were olaparib (5 µM or indicated dose), DNA-PKcsi (NU7441, 1 µM), Polθi (ART558, 10 µM). (**F**) HR quantification using the DR-GFP reporter in HT1080 cells after an ISceI- or Cas9-induced DSB, ± olaparib treatment (5 µM). Statistical analyses (**C–F**): Data represent three (D–F) or four (**C**) independent experiments, each the average of three technical replicates. Data are mean ± SEM. Statistical test, one way ANOVA with multiple comparison correction. ns: nonsignificant, *P <.05, **P <.01, ***P <.001, ****P <.0001.

Sequencing data from the ISceI reporter revealed an additional repair outcome with a clear MMEJ signature that was strongly suppressed by Polθ inhibition (“−9″ repair signature, Supplementary Fig. S1E). Notably, olaparib treatment had no effect on this repair outcome, suggesting that PARP1/2 inhibition may differentially regulate distinct MMEJ events. This prompted us to test whether PARPi promotes MMEJ across different DSB repair substrates. Cleavage by ISceI leaves 4 nucleotide 3′ overhangs. To generate blunt ends, we induced DSBs using Cas9 instead of ISceI (Fig. 4A). Using the same experimental timeline as with the ISceI system, we transiently transfected a plasmid expressing Cas9, a guide RNA targeting our reporter, and GFP (Fig. 4B). As expected, no fluorescence was detected without Cas9 transfection (Fig. 4C). Meanwhile, upon Cas9 DSB induction, the percentage of mCherry + cells decreased upon Polθ inhibition and increased upon DNA-PKcs inhibition (Fig. 4C). Interestingly, olaparib led to no measurable difference in Cas9-mediated MMEJ levels (Fig. 4C), a result that remained consistent across a range of olaparib doses (Fig. 4D). Similarly, double inhibition of PARP1/2 and Polθ had no effect compared to Polθi only (Fig. 4E).

To test whether olaparib differentially affects ISceI- and Cas9-mediated DSBs due to the short overhangs created by ISceI, we used a previously characterized Cas9-CtIP construct [79] to artificially generate overhangs at Cas9-induced breaks. However, like at blunt-end Cas9 breaks, olaparib had no effect on MMEJ activity at these sites (Supplementary Fig. S4A). We next hypothesized that olaparib would affect the repair pathway choice differently at these types of breaks. For instance, blunt-ended DSBs could be preferentially repaired by NHEJ, rather than MMEJ, when HR is suppressed. This hypothesis predicts that MMEJ would not be increased at Cas9-induced breaks upon suppression of HR. However, we found that *BRCA2*-KO cells displayed markedly higher (∼6 fold) MMEJ levels than control cells following induction of a blunt-ended Cas9 cut (Supplementary Fig. S4B). Thus, we cannot conclude whether the observed differences between ISceI- and Cas9-mediated DSBs arise from the break structure itself or from other factors, such as distinct enzymatic kinetics or steric hindrance.

Given that the increase in MMEJ is dependent on HR, we asked whether HR is differentially regulated by PARPi at ISceI and Cas9 breaks, using the well-established DR-GFP reporter [75] to measure HR activity. We established stable HT1080 cells carrying the DR-GFP reporter, transfected them with ISceI or Cas9 and treated them with olaparib, following the same timeline as for the MMEJ reporter. We found that PARPi leads to a significant decrease in HR activity at ISceI breaks but not at Cas9 breaks (Fig. 4F). While it remains unclear what causes the difference between the two methods of DSB induction, these results indicate that the effect of PARPi on MMEJ is likely a direct consequence of its effect on HR.

Overall, these data suggest that inhibition of PARP1 prevents a subset of DSBs from being repaired by HR, thereby driving them towards MMEJ. Nonetheless, in all the experimental settings tested, PARP1/2 inhibition never led to decreased MMEJ levels, suggesting that PARP1 and 2 are dispensable for MMEJ repair of intrachromosomal breaks.

### PARP1 and PARP2 are dispensable for MMEJ repair at deDSBs

**Figure 5.**
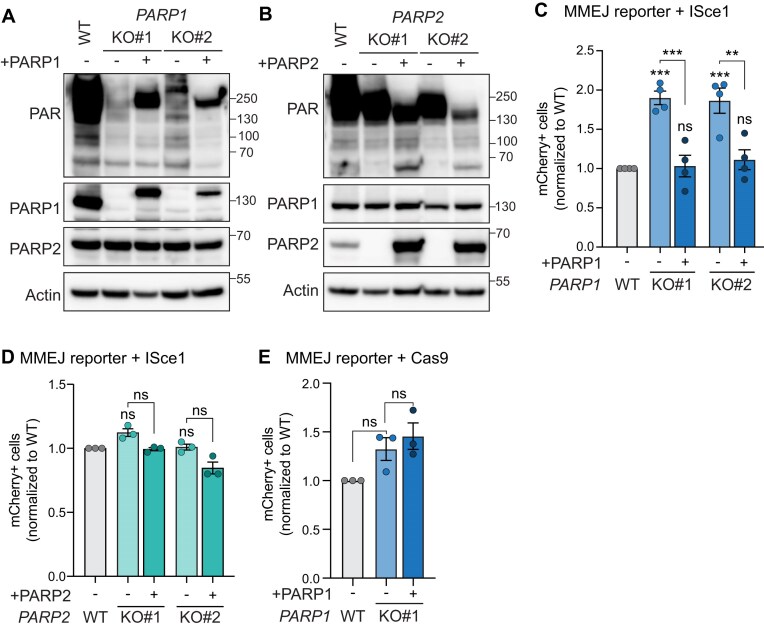
PARP1 and PARP2 are dispensable for MMEJ at deDSBs. (**A, B**) Immunoblot of Actin, PARP1, PARP2, and PAR in HT1080 cells and isogenic *PARP1*-KO with or without complemented GFP-PARP1 (**A**) or *PARP2*-KO with or without complemented PARP2 (**B**). (**C, D**) MMEJ quantification using the reporter with ISceI cut (from Fig. 1A and B) in HT1080 parental cells and indicated isogenic *PARP1*-KO with or without complemented GFP-PARP1 (**C**) or *PARP2*-KO with or without complemented PARP2 (**D**). Values are normalized to wild-type DMSO. Drug used was olaparib (5 µM). (**E**) MMEJ quantification using reporter with Cas9 cut (from Fig. 4A and B) in HT1080 cells and isogenic *PARP1*-KO cells with or without complemented GFP-PARP1. Values are normalized to wild-type DMSO. Statistical analyses (**C–E**): Data represent four (**C**) or three independent (**D, E**) experiments, each the average of three technical replicates. Data are mean ± SEM. Statistical test, one way ANOVA with multiple comparison correction. ns: nonsignificant, **P <.01, ***P <.001.

PARP1 was originally identified as a key factor in MMEJ and is widely considered a central promoter of this pathway [39, 69, 85]. Consistently, we and others have shown that PARP1/2 inhibition and PARP1 knockdown reduce MMEJ following telomere deprotection in mouse embryonic fibroblast (MEF) cells [44, 61]. However, our data challenge this well-established view, suggesting that PARP1/2 may be dispensable for MMEJ, at least in the context of deDSBs in human cells. Given this contradiction with the prevailing model of MMEJ, we sought to rigorously test whether PARP1 and/or PARP2 are truly dispensable for MMEJ repair of blunt or overhang breaks. To this end, we complemented *PARP1*-KO and *PARP2*-KO clones with exogenous GFP-PARP1 and PARP2, respectively (Fig. 5A and B). While the *PARP2*-KO clones easily re-expressed PARP2 (Fig. 5B), GFP-PARP1 expression in *PARP1*-KO clones was variable and below wild-type levels (Fig. 5A). Similarly, PARylation was not fully restored, mirroring GFP-PARP1 protein expression (Fig. 5A). To restrict our analysis to cells with higher PARP1 re-expression, we took advantage of the fact that PARP1 was fused to GFP and gated our flow cytometry analysis of MMEJ on cells with “high” GFP expression. We compared MMEJ levels between *PARP1* or PARP*2* knock-out and complemented cells following ISceI transfection. Consistent with the data showing that PARPi increases MMEJ (Fig. 1C), *PARP1*-KO clones showed elevated levels of MMEJ compared to control cells, which were rescued by exogenous PARP1 expression (Fig. 5C). Notably, the cell cycle of these clones remains unaltered (Supplementary Fig. S5A and B), further confirming that the observed increase in MMEJ upon PARP1 suppression is not a consequence of cell cycle perturbations. In contrast, *PARP2*-KO clones showed similar MMEJ levels to control cells, and complementation with exogenous PARP2 did not have any effect (Fig. 5D). These data parallel our finding that the absence of PARP2 does not change olaparib’s effect on MMEJ (Fig. 2D).

Finally, we assessed the effect of *PARP1* and *PARP2* knockout complementation on Cas9-induced MMEJ. Since both the PARP1 and Cas9 plasmids expressed GFP, we could not analyse mCherry + cells in the GFP + population in this experiment; instead, we measured the total mCherry + population. Similarly, we could not overcome the low expression of PARP1 in clone 2 by analysing specifically the high Cas9-GFP + cells. Therefore, we limited our analysis to clone 1. Consistent with our previous data, we found that neither *PARP1* knockout nor its rescue influenced MMEJ at Cas9-induced DSBs (Fig. 5E). Similarly, MMEJ levels remained unchanged in *PARP2*-KO and *PARP2*-KO complemented cells (Supplementary Fig. S5C). Altogether, our data demonstrate that PARP1 and PARP2 are dispensable for repair of both blunt and overhang deDSBs in human cells.

### MMEJ is active primarily during mitosis and, to a lesser extent, in S/G2

*In vitro* data have implicated a role for PARP1 in promoting MMEJ during interphase by competing with KU70/80 for DNA binding, facilitating synapsis formation at the break-site, and recruiting Polθ to the break-site [36, 55–58, 62]. While MMEJ is active in all cell cycle phases, it competes with NHEJ during G1 and with NHEJ and HR in S and G2 [26, 37, 40, 63–66], which would be expected to limit MMEJ activity during interphase. Conversely, two recent studies have shown that MMEJ is active during mitosis, when both NHEJ and HR are suppressed [72, 73]. At this stage, mitotic-specific factors RHINO and PLK1 promote the recruitment of Polθ to DSBs [72, 73].

**Figure 6.**
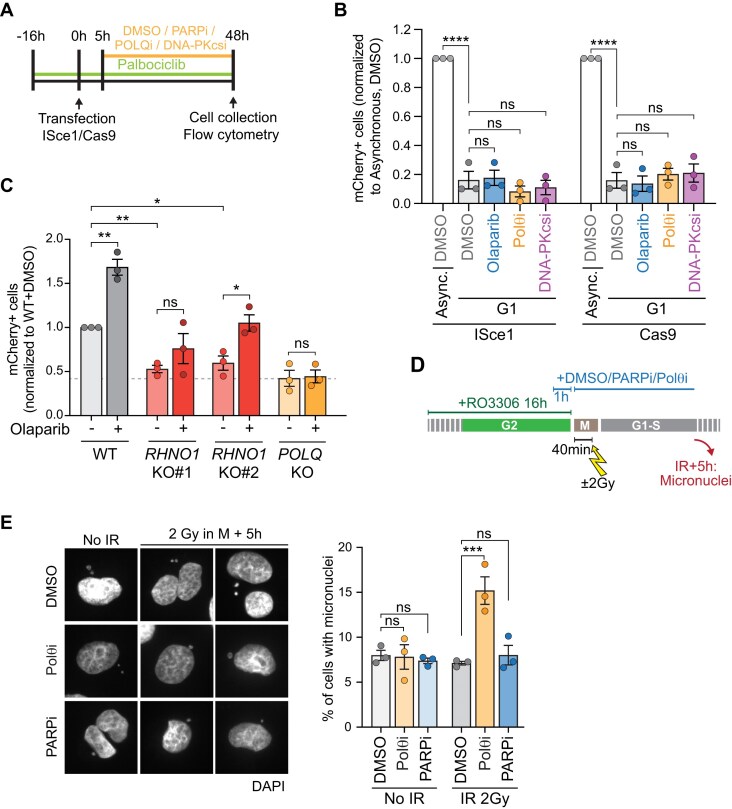
MMEJ is predominantly active in mitosis, when PARP1 is dispensable for DSB repair. (**A**) Experimental timeline for panel (B). Sixteen hours prior to transfection, cells were synchronized in G1 with palbociclib (250 nM), drugs were added 5 h after transfection and flow cytometry was performed 48 h later. A control was performed with the same timeline but without palbociclib synchronization. (**B**) MMEJ quantification using the MMEJ reporter with ISceI or Cas9 cut in asynchronous or G1-synchronized HT1080 cells. Drugs used: olaparib (5 µM), Polθi (ART558, 10 µM), DNA-PKcsi (NU7441, 1 µM). Data represent three independent experiments. (**C**) MMEJ quantification using the reporter with ISceI cut in HT1080 parental cells and indicated isogenic *RHNO1*-KO or *POLQ-*KO clonal lines, ± olaparib 5 µM. Data represent three independent experiments, each the average of three technical replicates. (**D**) Experimental timeline for panel (E). HT1080 cells are arrested in RO3306 (9 µM) for 16 h DMSO (0.1%), olaparib (5 µM), or Polθi (ART558, 10 µM) were added 15 h after RO3306. After 16 h in RO3306, RO3306 was removed and cells were maintained in respective DMSO, olaparib, or Polθi treatment. Cells were irradiated (2 Gy) 40 min after RO3306 release and fixed for micronuclei staining 5 h later. (**E**) Representative images (left) and quantification (right panel) of IF from HT1080 cells from panel (D) in cells 5 h post irradiation compared to no irradiation. Cells were stained for DAPI and the percentage of cells with micronuclei was quantified. Data represent three independent experiments. For each replicate, at least 300 cells were analysed. Statistical analyses for panels (B), (C), and (E): Statistical test, one way ANOVA with multiple comparison correction. ns: nonsignificant, *P <.05, **P <.01, ***P <.001, ****P <.0001.

We therefore sought to determine during which stages of the cell cycle MMEJ is active at our reporter. To evaluate MMEJ activity in G1, we synchronized cells with palbociclib for 16 h prior to ISceI or Cas9 transfection (Fig. 6A and Supplementary Fig. S6A). In both cases, we detected only very low frequencies of mCherry + cells in the palbociclib-treated population (Fig. 6B). Importantly, these residual levels were neither decreased by Polθ inhibition nor increased by DNA-PKcs inhibition, indicating that MMEJ is not active at these breaks during G1. To verify that MMEJ levels are low across different repair outcomes, including those with shorter deletions, we sequenced the reporter after the ISceI cut and found that both the –29 and –9 repair products were indeed minimal during G1 (Supplementary Fig. S6B).

We next asked whether MMEJ at our reporter depends on RHNO1, a marker of mitotic MMEJ [72]. *RHNO1*-KO clones displayed markedly reduced MMEJ, barely higher than *POLQ*-KO clones, indicating that a significant fraction of MMEJ events occur during mitosis, with residual activity in S/G2 (Fig. 6C ). Although olaparib treatment led to an increase in MMEJ, this effect was significant in only one of the two clones and did not recapitulate the magnitude of the increase observed in WT cells (Fig. 6C) . These results suggest that the PARPi-mediated increase in MMEJ reflects DSB repair activity taking place during both S/G2 and mitosis.

Together, our data indicate that PARP1/2 inhibition may prevent HR repair at certain breaks, diverting them toward MMEJ during S/G2 and mitosis. While we observed no MMEJ activity during G1, PARP1 appears dispensable for MMEJ at deDSBs in S/G2 and mitosis. Given that MMEJ is the only pathway active during mitosis, we next tested whether PARP1 is truly dispensable for DSB repair during this phase of the cell cycle.

### PARP1/2 are dispensable for DSB repair in mitosis

First, we assessed DSB repair in mitosis by staining for γH2A.X in phosphorylated Histone H3 serine 10+ (pH3S10+) cells following irradiation, as performed in [72] (experimental timeline depicted in Supplementary Fig. S6C). As previously described [72], γH2A.X foci significantly decreased 5 h post-irradiation in control cells, while they persisted in Polθi-treated cells, signifying that mitotic DSB repair relies on Polθ (Supplementary Fig. S6D and E). Treatment with olaparib led to higher levels of γH2A.X 1 h after irradiation and, while the damage signal did not fully return to baseline at 5 h, it was significantly decreased compared to 1 h, indicating that repair can occur in the presence of olaparib. It is unclear, however, whether the repair itself is delayed due to the higher level of damage at 1 h, or if olaparib could affect the dynamics of γH2A.X itself rather than repair.

To directly assess repair rather than γH2A.X signalling, we irradiated cells in mitosis and monitored micronuclei formation during the subsequent G1 (Fig. 6D), as these structures arise from unrepaired mitotic DSBs [96]. This approach also avoids prolonged mitotic arrest, providing a more physiological context. As previously reported, Polθ inhibition increased the number of micronuclei in G1 following irradiation [72]. Conversely, treatment with olaparib had no effect on the number of observed micronuclei (Fig. 6E), indicating that PARP1 is indeed dispensable for DSB repair during mitosis.

Together, these results demonstrate that MMEJ is not active at deDSBs during G1, but rather acts during mitosis, with limited activity during S/G2. Importantly, both mitotic and S/G2 MMEJ proceed independently of PARP1 and PARP2.

## Discussion

Through mechanistic and functional assays, we demonstrate that PARP1 and 2 are dispensable for MMEJ repair of deDSBs. Initial studies suggested that MMEJ activity depends on PARP1 [55–61, 97]. However, recent data indicate that certain alternative end-joining mechanisms involving PARP1 function independently of Polθ [93, 97]. Consistent with our findings, recent studies also suggest that PARP1 may not be essential for Polθ-dependent MMEJ [98, 99]. When genome-wide breaks were induced at sites with different chromatin contexts, PARP1 primarily promoted MMEJ repair at heterochromatic sites, suggesting that chromatin context may influence its role in MMEJ [98]. Additionally, G1-specific DSBs in NHEJ-deficient cells have been observed to undergo repair by Polθ in the S-G2/M phase without requiring PARP1 [99].

We found that MMEJ is inactive at deDSBs during G1, consistent with prior observations that Ku70 suppresses MMEJ in this phase [93]. In contrast, MMEJ activity was significantly reduced upon RHINO suppression, indicating that MMEJ mainly functions during mitosis, although some activity may occur during S and G2. During mitosis, PLK1-activated RHINO recruits Polθ to DSBs [72, 73], a function previously attributed to PARP1 [36, 62].

Conversely, the earlier studies that described PARP1 as essential to promoting MMEJ were done in the context of DNA-PK suppression, in which MMEJ is likely reactivated in G0/G1 [56, 92, 93]. These include fusions of deprotected telomeres in Ku80-KO MEFs, which occur in G1 [36, 44]. Although we found no MMEJ activity during G1, even after DNA-PKcs inhibition, we could not test our MMEJ reporter upon Ku deletion, since Ku loss is lethal in human cells [100]. It remains to be determined whether MMEJ is observed during G1 in Ku-deficient mice cells because of the loss of Ku or due to species differences. Therefore, we propose that in cells with intact pathways, MMEJ repairs deDSBs mostly during mitosis, when activated RHINO can recruit Polθ, rendering PARP1 dispensable. On the other hand, in contexts where MMEJ is active during G0/G1, either at deprotected telomeres in Ku-depleted MEFs cells, or during class-switch recombination in murine B-cells, MMEJ may depend on PARP1 for the recruitment of Polθ. Similarly, factors required for MMEJ may vary depending on the cell-cycle context. Since PARP1 was shown to recruit XRCC1 to breaks [5, 55, 101], it would be interesting to determine whether mitotic MMEJ uses XRCC1-LIG3 for the ligation step or, conversely, relies on LIG1. Likewise, Polθ is a target for PARylation [89, 102]. It will be interesting to tease apart the function of this PARylation during different phases of the cell cycle.

Similarly, most DSBs in unchallenged cells arise from collapsed replication forks. Thus, single-ended DSBs are likely more physiological than deDSBs, especially in the context of PARP inhibitors. It will be critical to determine which factors promote MMEJ at these types of breaks.

We also found that PARPi treatment led to an increase in MMEJ levels following ISceI-mediated intrachromosomal breaks. This increase was specifically dependent on PARP1, not PARP2. These findings illustrate that PARP1 and PARP2 have distinct effects on MMEJ, likely due to differential roles in competing pathways. Indeed, several labs have reported divergent functions between PARP1 and PARP2 on DDR, class switch recombination, PARylation, and different affinities for DSBs [8, 59, 103, 104].

Interestingly, we observed that PARP1 suppression only increased MMEJ levels when the DSB was induced by ISceI, not Cas9. This differential MMEJ response could be attributed to several reasons. For instance, it could be due to differential enzyme recruitment kinetics and the efficiency of DSB induction. Indeed, we consistently observed higher overall MMEJ levels following Cas9 break induction compared to ISceI (data not shown). Similarly, the mechanisms regulating endonuclease removal from break sites likely differ and could lead to differential effects. It is possible that PARPi-treated Cas9 breaks lead to mild increases in MMEJ levels that are not detected by our system. Conversely, it is possible that the difference between ISceI and Cas9 lies in the type of break and, therefore, could be due to differential DDR responses at blunt vs overhang DSBs. The increase following PARPi treatment from ISceI breaks may be due to effects on repair factors that process and recognize the 3′ overhangs. Indeed, PARP1 has been implicated in regulating resection at DSBs, though existing data remain contradictory. PARP1 has been shown to promote resection for MMEJ repair in amplicon sequencing experiments following the repair of a Cas9-induced cut [105]. In contrast, others have shown that PARP1 antagonizes or limits resection at breaks [106, 107]. Concordant with all this data, PARP1 can PARylate and/or interact with many proteins known to either promote or prevent resection, including BRCA1, MRE11, KU80, and DNAPKcs, all of which can change the balance in DSB repair pathway determination [6, 107–109]. Alternatively, or concurrently, PARP1 suppression may cause an overloading of the HR pathway, reducing its availability to repair ISceI-induced breaks. In contrast, HR may remain efficient at Cas9-mediated breaks, despite the limiting context. We observed that PARPi suppresses HR at ISceI but not Cas9 breaks. Although this experiment explains why PARPi mediates an increase in MMEJ at ISceI breaks only, it remains compatible with either a direct inhibition of HR by PARPi or an indirect overload of the pathway.

Finally, our findings strengthen the rationale for a combined therapeutic approach using PARPi and Polθi in HRD cancers. If PARP1 was required for MMEJ, then further inhibition of MMEJ should be futile. Our findings show that, on the contrary, direct inhibition of MMEJ is likely to work synergistically with PARPi. Moreover, translational data show that Polθ may contribute to PARPi resistance in HRD cancer cells. Indeed, some *BRCA* reversion mutations in diagnosed HRD tumours contain an MMEJ repair signature [31, 32]. We observed increased MMEJ levels of ISceI-induced DSBs following clinically used PARPi drugs in both HR-proficient and *BRCA2*-mutated cells (Fig. 1). If PARPi inadvertently leads to an upregulation of Polθ-mediated MMEJ for certain types of breaks, which can, in turn, lead to PARPi resistance, then blocking Polθ activity could also act in preventing resistance and thereby lengthening progression-free survival in patients taking PARPi.

## Supplementary Material

gkaf1437_Supplemental_File

## Data Availability

Sequencing data of the MMEJ reporter are available through NCBI SRA (https://dataview.ncbi.nlm.nih.gov/object/PRJNA1238271). All other data are available upon request from the corresponding author.
