# Supplementary material for: PARP1 and PARP2 are dispensable for DNA repair by microhomology-mediated end-joining at double-ended DSBs
Source: Nucleic Acids Res. 2026 Jan 6;54(1):gkaf1437. doi: 10.1093/nar/gkaf1437 (PMC12774638; doi:10.1093/nar/gkaf1437)
Supplement: gkaf1437_Supplemental_File [file gkaf1437_supplemental_file.pdf]

Supplementary materials to:

PARP1 and PARP2 are dispensable for DNA repair by microhomology-mediated end-joining at double ended DSBs.

Raquel Ortega[1,2], Erin Taylor[1], Sophie M. Whitehead[1], Thomas Danhorn[3,4], Benjamin G. Bitler[2,†,*], Nausica Arnoult[1,†,*]

[1] Department of Molecular, Cellular, and Developmental Biology, University of Colorado Boulder, Boulder, Colorado, 80309, USA
[2] Division of Reproductive Sciences, Department of Obstetrics and Gynecology, University of Colorado Denver, Anschutz Medical Campus, Aurora, Colorado, 80045, USA
[3] University of Colorado Cancer Center, University of Colorado Anschutz Medical Campus, Aurora, Colorado, 80045, USA
[4]Department of Biomedical Informatics, University of Colorado Anschutz Medical Campus, Aurora, Colorado, 80045, USA

† Joint Authors

* To whom correspondence should be addressed:
Nausica Arnoult, Tel: +1 (303) 735 7148; Email: nausica.arnoult@colorado.edu

* Correspondence may also be addressed to:
Benjamin G. Bitler, Tel: +1 (303) 724-0574; Email: benjamin.bitler@cuanschutz.edu

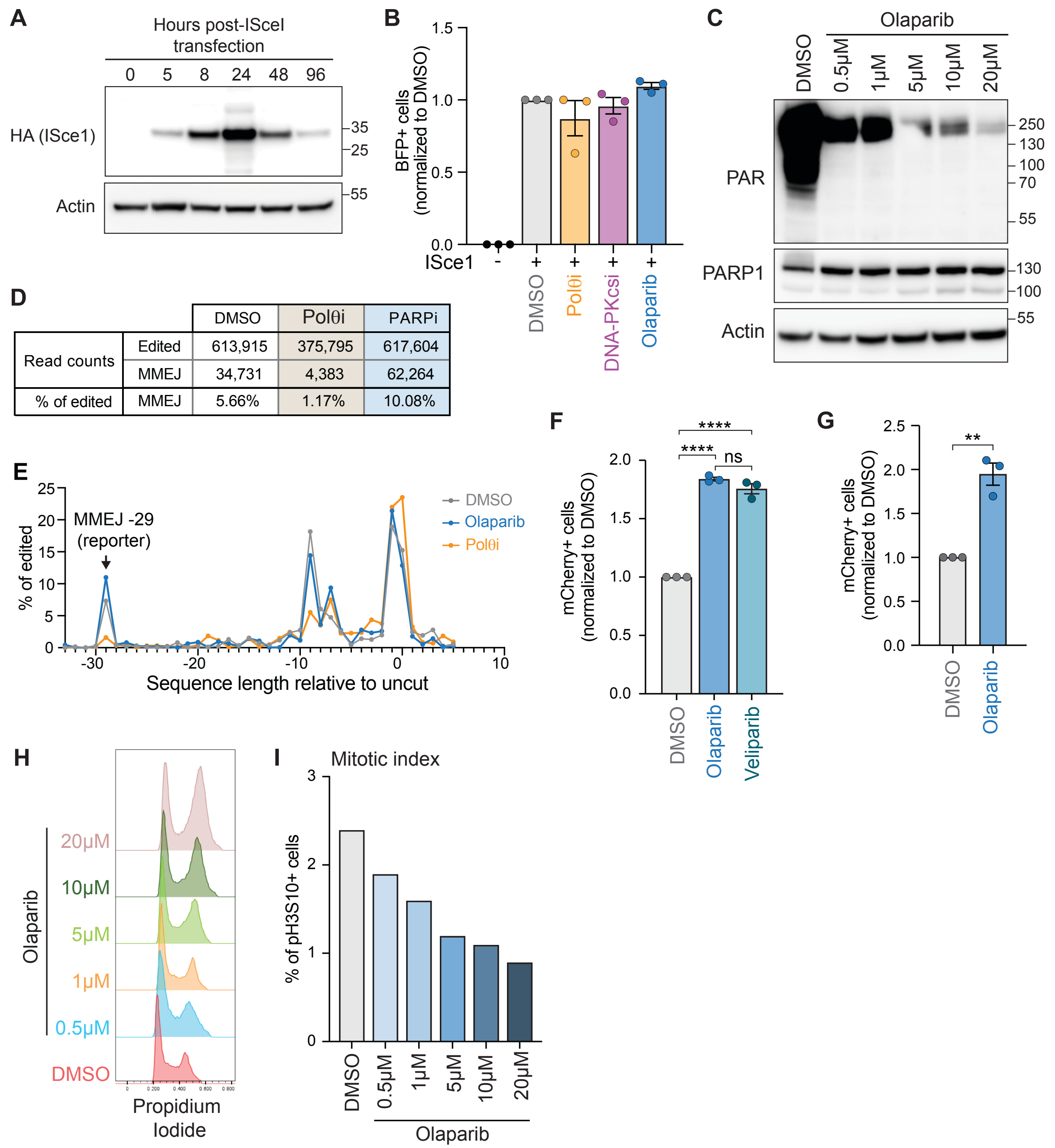

		DMSO	Polθi	PARPi
Read counts	Edited	613,915	375,795	617,604
	MMEJ	34,731	4,383	62,264
% of edited	MMEJ	5.66%	1.17%	10.08%

Supplementary Figure 1.

(**A**) Immunoblot of HA-IScel and actin in HT1080 cells at the indicated time after IScel transfection. (**B**) BFP+ (IScel+) quantification from Figure 1C in HT1080 cells. Values are normalized to DMSO. (**C**) Immunoblot of PARP1, Actin, and PAR in dose escalated olaparib

HT1080 cells. (**D-E**) Amplicon sequencing from the MMEJ reporter and timeline from Figure 1A-B in HT1080 cells following DMSO, Polθi (ART558, 10 μM), or olaparib (5 μM) treatment. (D) Represent the number of exact sequence match for MMEJ reporter repair relative to all edited sequences. (E) Represents all edited products according to sequence lengths. mCherry+ product corresponds to the -29 nucleotides deletion. Some –29 repair products deviate slightly from the canonical MMEJ sequence, explaining the minor differences between panels (D) and (E). One replica. (**F**) MMEJ quantification using reporter and timeline from Figure 1A-B in HT1080 cells following olaparib (5 μM) or veliparib (5 μM) treatment. Values are normalized to DMSO. (**G**) MMEJ quantification using the reporter from Figure 1A but with olaparib addition 24 hours prior to transfection. (**H**) Flow cytometry cell cycle analysis of dose escalated olaparib cells using propidium iodide staining. (**I**) Flow cytometry mitotic index using pH3S10+ cells following dose escalated olaparib. Statistical analyses for (**F-G**): Data represent three independent experiments, each the average of three technical replicates. Data are mean ± SEM. Statistical test, one way ANOVA with multiple comparison correction (F) or t-test (G). ns: non-significant, **p<0.01, ****p<0.0001.

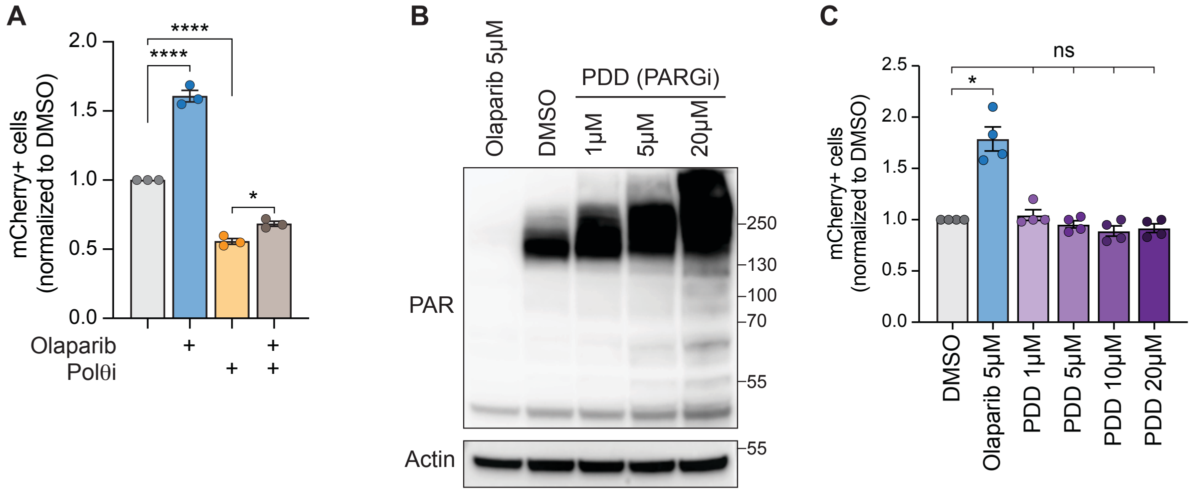

Supplementary Figure 2.

(**A**) MMEJ quantification using reporter and timeline from Figure 1A-B in HT1080 cells following olaparib (5 μM), Polθi (ART558, 10 μM) or combination. Values are normalized to DMSO. (**B**) Immunoblot of PAR and actin upon indicated drug treatment HT1080 cells. (**C**) MMEJ quantification using reporter and timeline from Figure 1A-B in HT1080 cells following olaparib (5 μM) or PARGi PDD (escalating doses) treatment. Values are normalized to DMSO. Statistical analyses for (**A, C**): Data represent three (A) or four (C) independent experiments, each the average of three technical replicates. Data are mean ± SEM. Statistical test, one way ANOVA with multiple comparison correction. ns: non-significant, *p<0.05, ****p<0.0001.

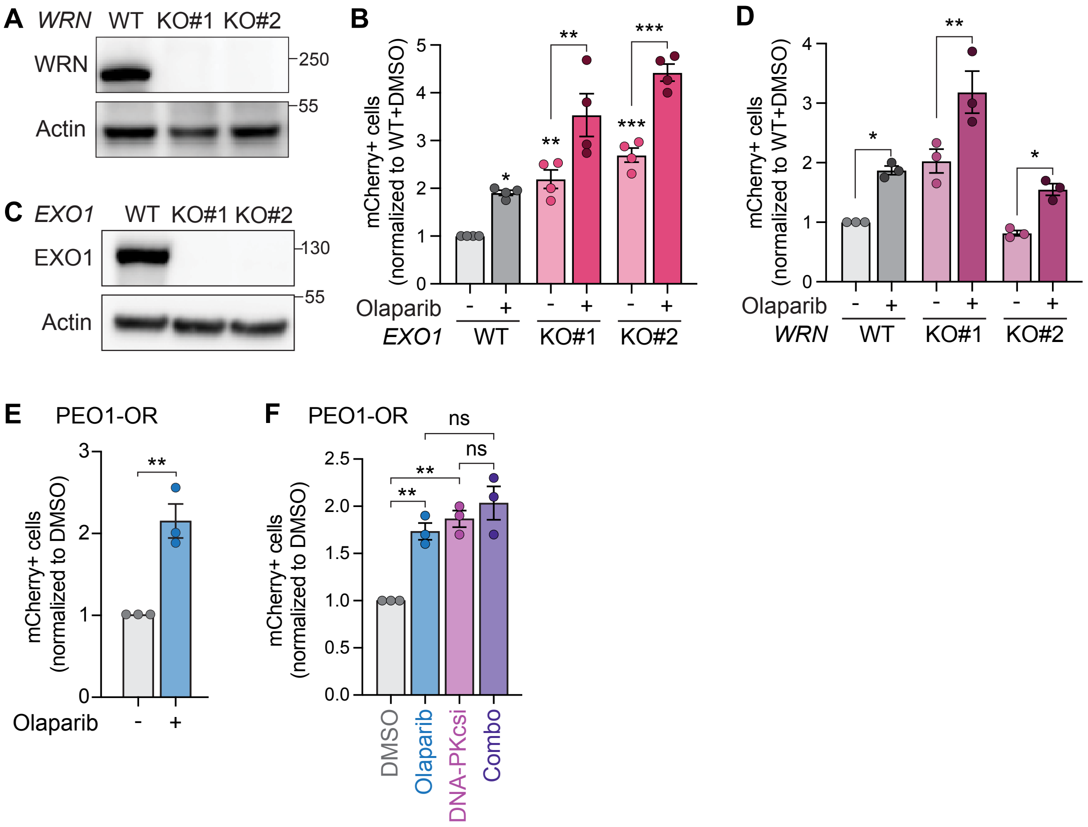

Supplementary Figure 3.

(**A**) Immunoblot of Actin and WRN in parental HT1080 cells and isogenic *WRN*-KO clonal cells. (**B**) MMEJ quantification in HT1080 parental cells and *WRN*-KO clones, ±olaparib (5µM). Values are normalized to wild-type DMSO. (**C**) Immunoblot of Actin and EXO1 in parental HT1080 cells and isogenic *EXO1*-KO clones. (**D**) MMEJ quantification in HT1080 parental cells and isogenic *EXO1*-KO clones, ±olaparib (5µM). Values are normalized to wild-type DMSO. (**E**) MMEJ quantification in PEO1-OR (olaparib-resistant) cells following olaparib treatment (5µM). Values are normalized to DMSO. (**F**) MMEJ quantification in PEO1-OR cells following olaparib (5 µM), DNA-PKcsi (NU7441, 2.5 µM), or combo. Statistical analyses for (**B, D, E, F**): Data represent three (D, E, F) or four (B) independent experiments, each the average of three technical replicates. Data

are mean ± SEM. Statistical test, one way ANOVA with multiple comparison correction (B, D, F) or t-test (E). ns: non-significant, *$p<0.05$, **$p<p<0.01$, ***$p<0.001$.

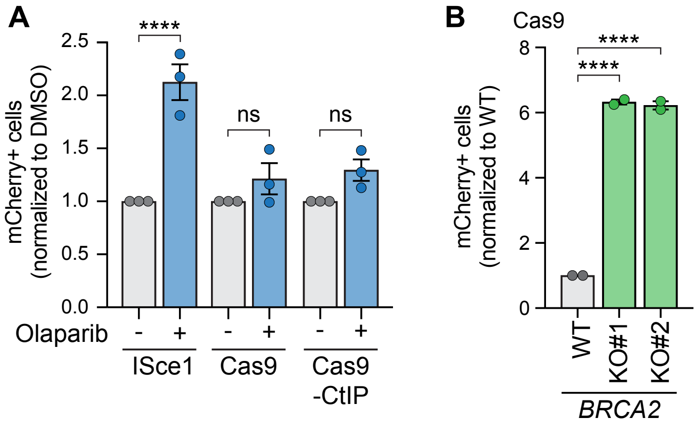

Supplementary Figure 4.

(**A**) MMEJ quantification in HT1080 parental cells, with DSB induced with ISceI, Cas9, or Cas9-CtIP, ±olaparib (5μM). Values are normalized to wild-type DMSO. (**B**) MMEJ quantification in HT1080 parental cells and *BRCA2*-KO clones, after a Cas9-mediated DSB. Values are normalized to wild-type. Statistical analyses for (**A-B**): Data represent three (A) or two (B) independent experiments, each the average of three technical replicates. Data are mean ± SEM. Statistical test, one way ANOVA with multiple comparison correction. ns: non-significant, ****$p<0.0001$.

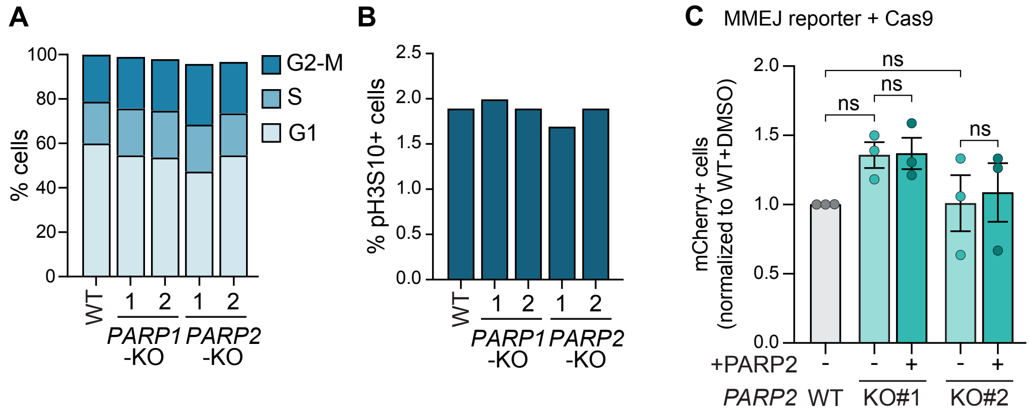

Supplementary Figure 5.

(**A**) Flow cytometry-based cell cycle analysis using propidium iodide (PI) of cells used in (C), Figure 2C-D, and Figure 5C-E. (**B**) Flow cytometry mitotic index using pH3S10+ of cells used in (C), Figure 2C-D, and Figure 5C-E. (**C**) MMEJ quantification using the reporter with Cas9 cut (from Figure 4A-B) in HT1080 cells and isogenic *PARP2*-KO cells with or without complemented *PARP2*. Values are normalized to wild-type DMSO. Statistical analyses for (**C**): Data represent three independent experiments, each the average of three technical replicates. Data are mean ± SEM. Statistical test, one way ANOVA with multiple comparison correction. ns: non-significant.

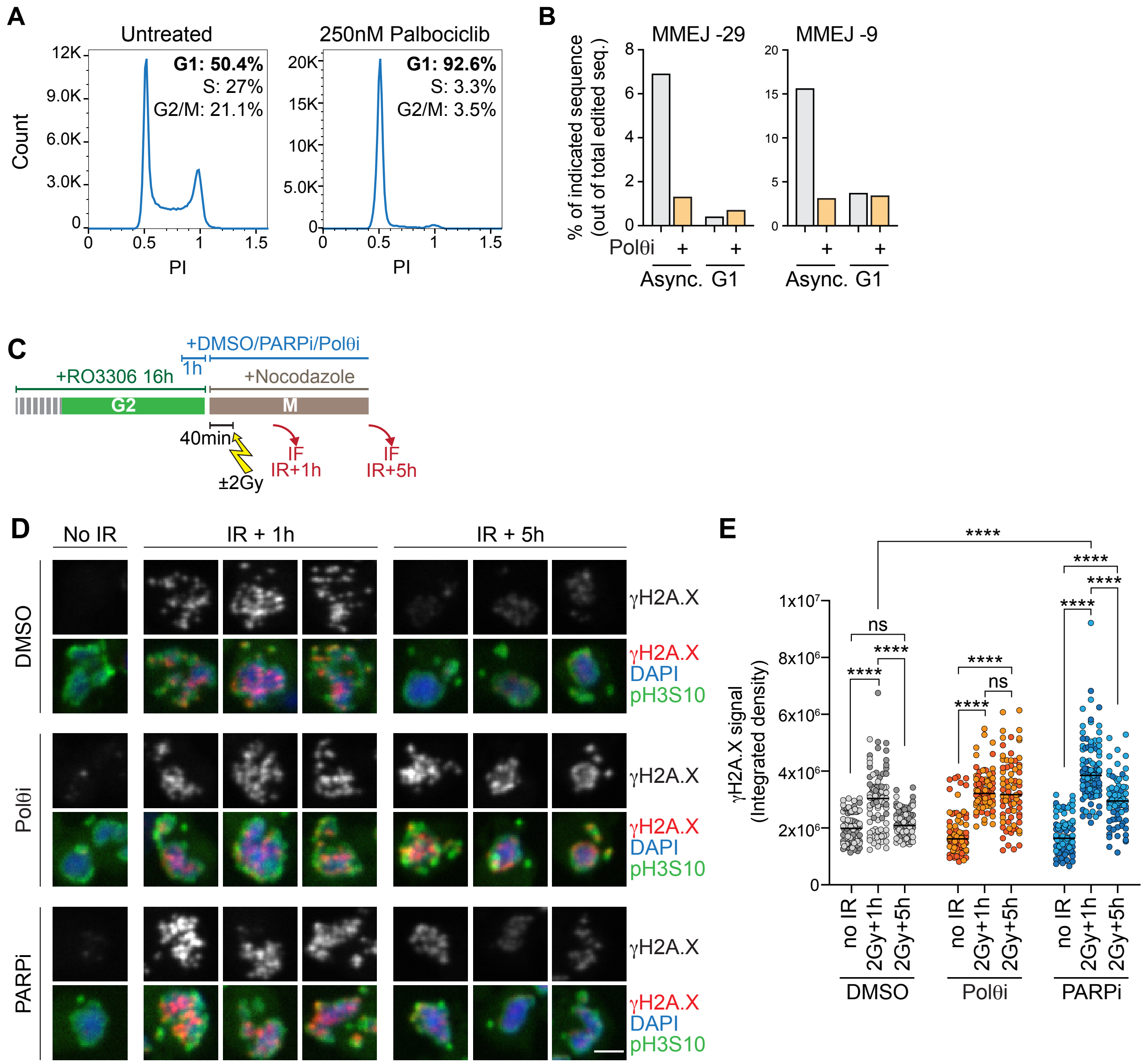

Supplementary Figure 6.

(**A**) Flow cytometry-based cell cycle analysis using propidium iodide (PI) of cells used in (B) and Figure 6B. (**B**) Amplicon sequencing of asynchronous and G1-synchronized HT1080 cells after IsceI-induced DSB in the MMEJ reporter, ±olaparib (5μM). Data represent % of sequences with the indicated deletion, corresponding to two different MMEJ repair outcomes. One replica. (**C**) Experimental timeline of (D) and (E). HT1080 cells were arrested in RO3306 (9 μM) for 16 hours. DMSO (1%), olaparib (5 μM), or Polθi (ART558, 10 μM) were added 15 hours after RO3306. After

16 hours in RO3306, cells were released into nocodazole (10 ng/ml) and maintained in respective DMSO, olaparib, or Polθi treatment. Forty minutes after RO3306 wash, cells were irradiated (2 Gy), and samples collected 1 hour and 5 hours later. (**D-E**) Representative images (D) and quantification (E) of IF from HT1080 cells from (C) at 1 hour and 5 hours post irradiation compared to no irradiation. Cells were stained for pH3S10, γH2A.X, and DAPI. Scale bar: 10μM. γH2A.X intensity was quantified in pH3S10+ cells. Statistical analyses for (**E**): Data represent two independent experiments, with at least 35 cells per replicate. Data are mean ± SEM. Statistical test, one way ANOVA with multiple comparison correction. ns: non-significant, ****$p<0.0001$.
